# 3D Finite Element Model for Writing Long-Period Fiber Gratings by CO_2_ Laser Radiation

**DOI:** 10.3390/s130810333

**Published:** 2013-08-12

**Authors:** João M. P. Coelho, Marta Nespereira, Manuel Abreu, José Rebordão

**Affiliations:** 1 Laboratory of Optics, Lasers and Systems, Faculty of Sciences, University of Lisbon, Campus do Lumiar, Estrada do Paço do Lumiar, 22, Building D, 1649-038 Lisboa, Portugal; E-Mails: mcnespereira@fc.ul.pt (M.N.); maabreu@fc.ul.pt (M.A.); jmrebordao@fc.ul.pt (J.R.); 2 Institute of Biophysics and Biomedical Engineering, Faculty of Sciences, University of Lisbon, Faculdade de Ciências da Universidade de Lisboa, Campo Grande, Lisboa 1749-016, Portugal

**Keywords:** laser processing, long-period fiber gratings, finite element modeling, fiber-based sensors, refractive-index modulation, thermo-mechanical processes

## Abstract

In the last years, mid-infrared radiation emitted by CO_2_ lasers has become increasing popular as a tool in the development of long-period fiber gratings. However, although the development and characterization of the resulting sensing devices have progressed quickly, further research is still necessary to consolidate functional models, especially regarding the interaction between laser radiation and the fiber's material. In this paper, a 3D finite element model is presented to simulate the interaction between laser radiation and an optical fiber and to determine the resulting refractive index change. Dependence with temperature of the main parameters of the optical fiber materials (with special focus on the absorption of incident laser radiation) is considered, as well as convection and radiation losses. Thermal and residual stress analyses are made for a standard single mode fiber, and experimental results are presented.

## 1. Introduction

Long-period fiber grating, or LPFG, play an important role in the development of fiber-based sensors in several areas of engineering. In the field of sensing systems, they are well suited to measure mechanical quantities: they can be applied as structural bend sensors, temperature sensors, axial strain sensors, refractive index sensors and biochemical optical sensors [1–7].

A LPFG can be considered a particular type of Fiber Bragg Grating, or FBG, in which the period of the index modulation, Λ, satisfies a phase matching condition between the fundamental core mode and a forward propagating cladding mode. This condition relates the resonant wavelength of the light into a particular cladding mode *m*, *λ*^m^_res_, the effective refractive index of the core, *n_eff_*_,_*_co_*, and the effective refractive indexes of the *m*th-cladding mode, *n^m^_eff_*_,_*_cl_*

(1) $$\lambda_{\text{res}}^{m}=\left(n_{\text{eff},co}-n_{\text{eff},cl}^{m}\right)\Lambda$$

These grating-based devices are produced by periodically creating a perturbation of the refractive indexes of the core and/or cladding along the length of the fiber. Typically the length of a FBG ranges from a few millimeters to about one centimeter, with modulation periods of a few dozens of microns. The length of a LPFG is of the order of a few centimeters with periods of hundreds of micrometers. LPFGs require therefore simpler fabrication processes and have lower costs, and show lower retro-reflection, higher sensitivity and robustness in sensing applications when compared with FBG [8].

A LPFG can be produced mechanically [9], chemically (etching) [10], by photonic processes (ultra-violet irradiation) [11] or thermally, either by applying an electric arc discharge [12] or a mid-infrared radiation (MIR) laser source [13,14]. Within the family of thermal techniques, MIR from CO_2_ lasers has guaranteed better predictability and repeatability [15].

Since the first reports by Davis *et al.* [13] and Akiyama *et al.* [14] on the use of a 10.6 μm wavelength laser beam emitted by a CO_2_ laser, different experimental methodologies have been used to write LPFGs [15]. The most common is, probably, using a static asymmetrical irradiation with a CW CO_2_ laser and a cylindrical lens focusing a laser line on the fiber. This method has the advantage of requiring a simpler setup and, although the irradiation occurs on just one of the sides of the fiber, the line-shaped beam reduces the writing asymmetric effect when compared with focused circular laser beams.

In parallel to the development of writing techniques, research on the physical mechanisms responsible by refractive index changes has progressed. Based on several experimental analysis, most of the existing work has considered that the main mechanism responsible for inducing a refractive-index change is the relaxation of internal stresses by the exposure to the laser radiation [16–21]. Taking this in consideration, several analytical models were developed, based mainly on solutions of the general heat conduction equation [22,23]. These models, although giving proven data on the necessary engineering parameters, are based on expressions that do not consider all the physical phenomena (e.g., convection losses) and assume several simplifications (e.g., they ignore the temperature dependence of glass parameters).

In the following sections, the different thermo-mechanical effects of the interaction between a MIR laser beam and a standard silica-based single mode optical fiber will be analyzed, both theoretically and experimentally, and a 3D finite element method (FEM) model, implemented in the COMSOL Multiphysics program, is described, considering temperature dependence of the main parameters, in particular the absorption of laser radiation by the fiber's material.

## 2. Thermo-Mechanical Model

**Figure 1.**
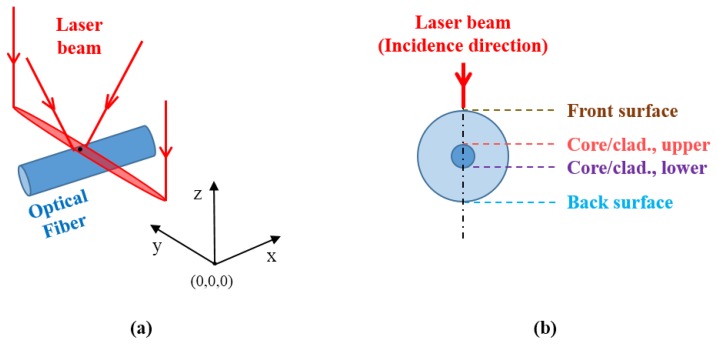
Schematics of (**a**) coordinates used in this work and (**b**) main physical interfaces. The origin of the reference system is in the middle of the laser line.

Considering a silica-based single mode optical fiber under tension irradiated by an elliptical 10.6 μm wavelength CO_2_ laser beam (Figure 1), two main phenomena must be considered: the thermal heating due to the interaction between the photons and the glass molecular structure and the stress due to the differences between a relatively low-viscosity doped silica core and a relatively high-viscosity pure silica cladding [16].

Differences between core and cladding thermal expansion coefficients and viscosity lead to residual thermal stresses and draw-induced residual stresses. In the case under study, these localized effects periodically induced along the fiber's length, can be responsible for the creation of the gratings. This effect is due to the refractive index change resulting from frozen-in viscoelasticity [17].

### 2.1. Theory

The refractive index change in a silica-based optical fiber can be approximated by the relation [17]:

(2) $$\Delta n\left(T\right)\approx -6.35\times 10^{-6}\sigma \left(T\right)$$

where *σ*(*T*) represents the overall (both thermal, *σ_T_*(*T*), and drawn-induced, *σ_x_*) residual stresses (in MPa) in the fiber's axial direction. According to Yablon [16], stresses in the other directions can be neglected.

The fiber is composed of a low viscosity, high thermal expansion coefficient core and a high viscosity, low thermal expansion coefficient cladding, the drawn-induced axial residual stresses act in opposition to the residual thermal stress. Either one can prevail or both can compensate each other.

The temperature distribution around a heat source can be obtained by solving the heat conduction problem. In what concerns the temperature variation with time, *t*, (transient regime) due to the action of a heat source *Q*(*x*,*y*,*z*,*t*), the resulting energy balance leads to the heat conduction equation:

(3) $$\left\{\frac{\partial \rho }{\partial t}+\nabla \cdot \left(\rho \vec{\nu }\right)\right\}\int Cp\;dT+\rho Cp\left(\frac{\partial T}{\partial t}+\vec{\nu }\cdot \nabla T\right)=\nabla \cdot K\nabla T+q(T)+Q(x,y,z,t)$$

being *v⃗* the velocity vector, *ρ* the density, *Cp* the specific heat, and *K* the thermal conductivity, these are the main parameters of the heated material. The factor *q*(*T*) quantifies the convective and radiative heat flux [22]:

(4) $$q\left(T\right)=h\left(T_{\inf}-T\right)+\varepsilon \sigma_{B}\left({T_{\text{amb}}}^{4}-T^{4}\right)$$

being *T_inf_* the external temperature, *T_amb_* the environment temperature, *h* the heat transfer coefficient, *σ_B_* the Stefan-Boltzmann constant and *ε* the surface emissivity.

When considering the condition of mass conservation, an isotropic material with *K* = *K*(*T*), and introducing the thermal diffusivity *k*, given by *K*/(*ρ Cp*), Equation (3) can be simplified to:

(5) $$\frac{\partial T}{\partial t}+\vec{v}\cdot \nabla T-k\left(\nabla \cdot \nabla T\right)+q(T)=\frac{Q(x,y,z,t)}{\rho C_{p}}$$

The power generation per unit volume of the material is given by *Q(x,y,z,,t)* and depends on the characteristics of the heat source. For a laser beam incident on a surface and propagating in the z direction:

(6) $$Q\left(x,y,z,t\right)=a_{T}.\left(1-R\right)I\left(x,y,z\right)g\left(t\right)$$

where *a_T_* is the attenuation coefficient of the material, *R* its reflectance and *I(r,t)* the irradiance. For continuous wave emission with a duration *τ*:

(7) $$g\left(t\right)=\{\begin{array}{c} 0,\text{if}\;t\leq 0\vee t>\tau \\ I(x,y,z),\text{if}\;0<t\leq \tau \end{array}$$

If the laser beam has an elliptical Gaussian distribution (at the surface being irradiated), then [24]:

(8) $$I(x,y,z)=\frac{8a_{T}P}{\pi d_{x}d_{y}}\exp\left[-2\left(\frac{x^{2}}{{d_{x}}^{2}}+\frac{y^{2}}{{d_{y}}^{2}}\right)\right]\cdot \exp(-a_{T}z)$$

where *d*_x_ and *d*_y_ are the dimensions of the ellipse's axis.

Solving Equation (5), the thermally-induced residual stresses, *σ*(*T*), can be obtained considering the constitutive equations for a linear isotropic thermoelastic material and the stress tensor obtained [25].

The residual axial elastic stresses in the cladding and core, *σ_cl_* and *σ_co_*, respectively, resulting from a draw tension *F*, over the equivalent cross-sectional areas *A_cl_* and *A_co_* can be obtained from [26]:

(9) $$\sigma_{x,cl}=\frac{F}{A_{cl}}\left(\frac{A_{co}E_{co}}{A_{co}E_{co}+A_{cl}E_{cl}}\right)$$

and:

(10) $$\sigma_{x,co}=F\left(\frac{E_{co}}{A_{co}E_{co}+A_{cl}E_{cl}}\right)$$

Besides stress-related refractive index change, localized heating can induce micro-deformation of the fiber and changes in the glass structure. The latter is likely to occur in the core for which the fictive temperature (glass structure doesn't change below the fictive temperature) is lower [18,27]. As reported, (e.g., [18]) for a Ge-doped core the fictive temperature ranges from 1,150 K to 1,500 K.

### 2.2. Physical Parameters

**Figure 2.**
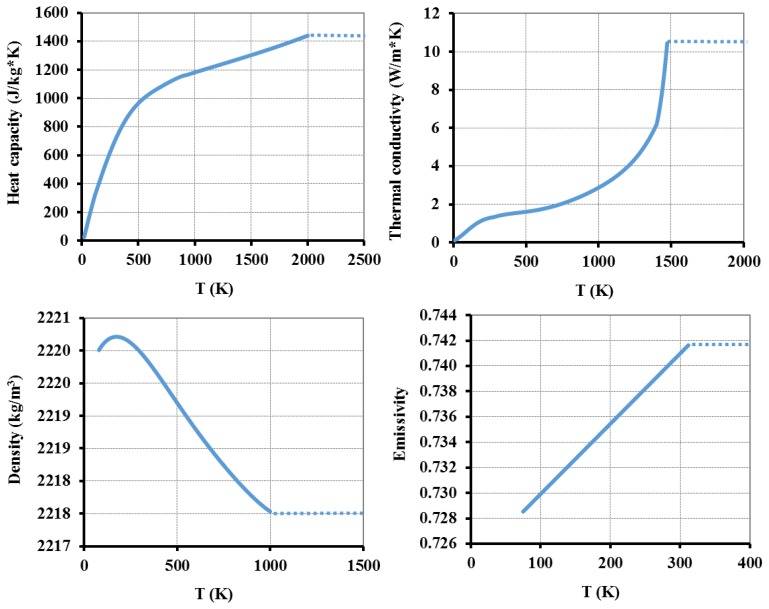
Variation of the main parameters used of Corning 7940 fused silica glass as defined in the COMSOL's materials library.

**Figure 3.**
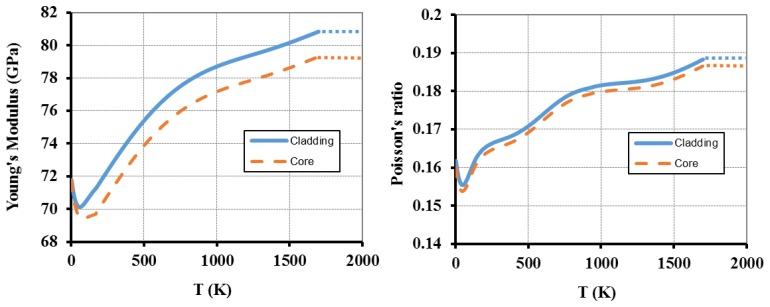
Variation of Young's module and Poisson's ratio for both fused silica (from COMSOL materials library) cladding and Ge-doped fused silica (extrapolated) core glasses.

**Figure 4.**
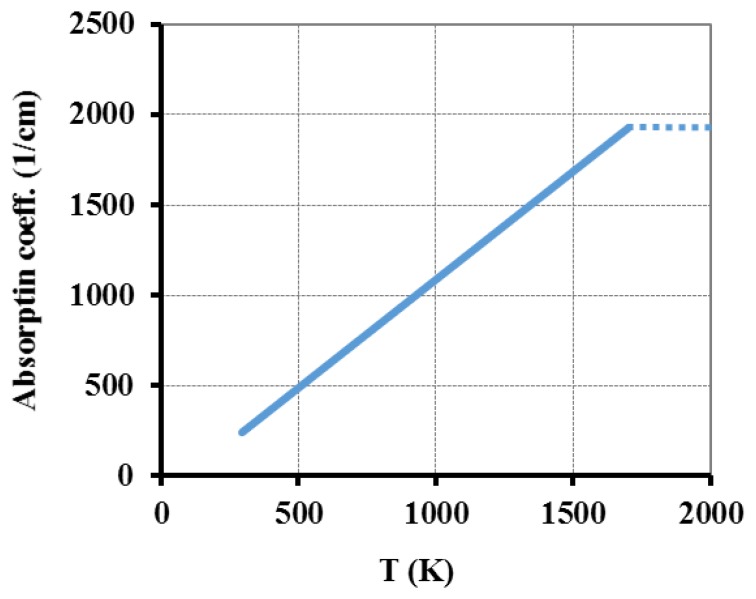
Absorption coefficient variation with temperature for fused silica glass accordingly with Equation (11).

The optical fiber is a Corning SMF-28 fiber (125 μm diameter pure fused silica cladding and 8.2 μm diameter core of 3.5 mol% Ge-doped SiO_2_) [28]. Figure 2, Figure 3 and Figure 4 show the change on the different parameters with temperature. For some parameters (shown in Figure 2), the temperature dependence was modelled using native COMSOL functions for a Corning fused silica glass (7940). The doping effect on most of the parameters was disregarded mainly because the Ge concentration in the fiber's core is very low [29]. However, for the Young's modulus and Poisson's ratio (Figure 3), we extrapolated the function behavior [30]. Furthermore, the heat transfer coefficient was considered *h* = 418.68 W·m^−2^·K^−1^ [22] and *R* = 0.15 [31].

Another factor to take in consideration is the material's absorption coefficient for the wavelength of interest. Accordingly with Tian [32] the absorption coefficient of fused silica, *a_T_*, for 10.59 μm for CO_2_ laser wavelength (*λ_1_*), within 298 K–2,073 K temperature range can be obtained by:

(11) $$a_{T}\left(T\right)=\frac{4\pi }{\lambda_{1}}\left[1.82\times 10^{-2}-10.1\times 10^{-5}\times \left(T-273\right)\right]$$

This equation was used and the corresponding curve is presented in Figure 4. In the plots presented in Figure 2, Figure 3 and Figure 4, the dotted lines represent the constant value that is assumed for higher temperature ranges.

### 2.3. Implementation

The physical problem was mathematically solved using the FEM model implemented using the COMSOL Multiphysics 3.5 program to create the transient heat conduction and (mechanical) stress-strain models under the conditions of this study. In order to introduce some of the complexity of stress-related issues regarding the processing of the optical fibers, the residual axial elastic stresses were implemented considering Equations (9) and (10) and the total resulting stress was obtained adding the thermally-induced residual stresses obtained with the program.

The implemented geometry consisted of a set of (concentric) cylinders with radius of curvatures accordingly with the characteristics of the core and cladding of the optical fiber described in the previous section. To avoid the influence of the external boundaries on the irradiated and analyzed zones, the overall length for the geometry was set as 11 mm. However, to reduce the computational load and loosen the mesh dimensions in zones not affected by the irradiation, the cylinders were implemented as three separate sets; the central one, where the laser incidence will be simulated, has a 700 μm length. Table 1 presents the 3D geometry data and the mesh statistics. Both outer boundary surfaces are defined as thermally isolated, being one of them fixed. The ambient temperature was considered to be 295 K and equal to the external temperature, *T_inf_* in Equation (4).

## 3. Experimental Methodologies

The experimental procedure was based on a simple point-by-point laser writing on the fiber characterized previously. The fiber was periodically moved (500 μm ± 1 μm grating period) along its axial direction with a linear translation stage (Thorlabs NRT100) and periodically irradiated (0.6 s ± 5 μs emission duration) by the beam emitted by a CW MIR CO_2_ laser source (Synrad 48-2); the maximum available power was 25 W.

**Figure 5.**
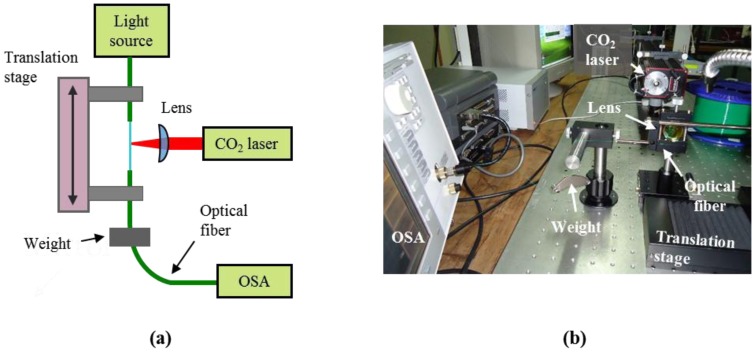
(**a**) Schematic and (**b**) photograph of the setup used for CO_2_ laser writing a LPFG on a Corning SMF-28 fiber.

A schematic and a photograph of the set-up are shown in Figure 5. A 50 mm focal length cylindrical lens focuses the initial 1.75 mm laser beam into a 0.15 mm × 1.75 mm (*r_x_* × *r_y_*) elliptical beam on the optical fiber. The dimensions of the beam on focus were measured through the knife-edge method, with an expected measurement error of ±5 μm [33].

In order to induce a constant strain to the fiber, a small weight (usually, several tenths of gram) is attached on one of the sides of the fiber, suspended. A broad band light source (Thorlabs S5FC1005S) and an optical spectrum analyzer (OSA, Agilent 86140B) allows monitoring the LPFG fabrication, while a fast camera (PCO SensiCAM), perpendicular to the irradiation axis, was used to optically visualize the process. The irradiated zones were analyzed through an optical microscope (Zeiss AxioScope A1) with maximum amplification of 1,000×.

## 4. Results and Analysis

**Figure 6.**
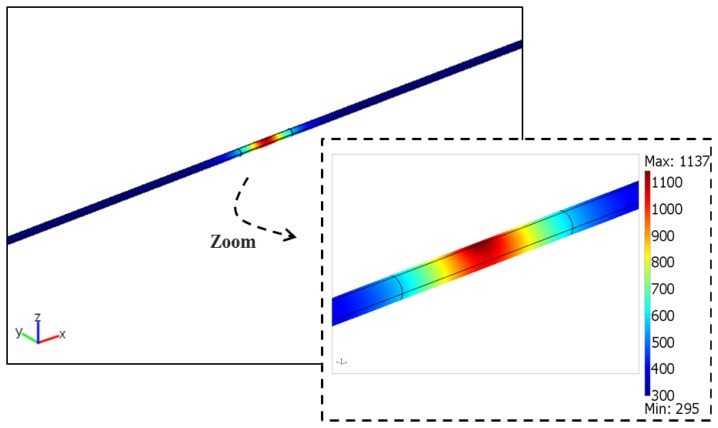
Temperature distribution in the implemented 3D geometry for the laser irradiation of an optical fiber (*P* = 6 W; *τ* = 0.6 s; *F* = 0.461 N). Color bar values are in K.

An example of the temperature distribution is shown in Figure 6, including a zoom view of the irradiated zone, for 6 W (±0.5 W) laser power, duration of 0.6 s (±1 ms) and 47 g (±0.5 g) weight (*F* = 0.461 N), the base parameters for this study, taking in consideration the experimental results.

**Figure 7.**
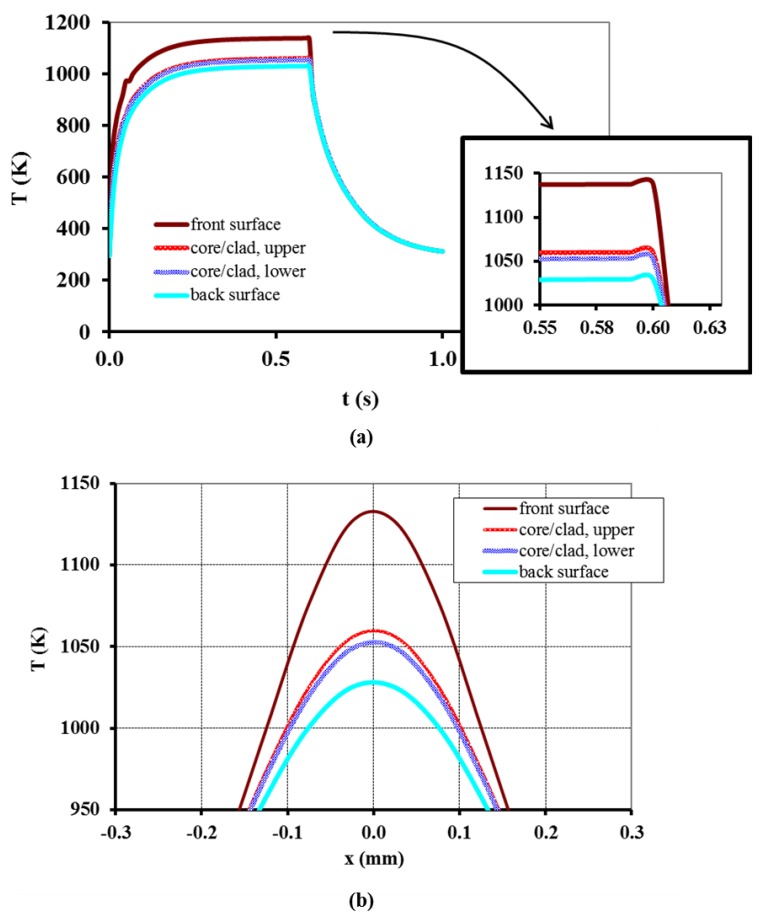
Plots of the temperature (**a**) evolution during laser irradiation and cooling and (**b**) distribution at the fiber's axial direction simulated for *t* = 0.6 s. (*P* = 6 W; *F* = 0.461 N).

Figure 7 shows the plots resulting from the simulation using Equation (5) under the conditions mentioned before for the irradiated front surface, core/cladding interfaces (upper and lower) and the back surface of the fiber (Figure 1), and *x* = *y* = 0 m. In Figure 7a, the duration of the simulation was made larger than the laser emission duration in order to visualize the cooling process. The plot in Figure 7b, representing the variation of temperature along the fiber's axial direction, shows that the temperature was slightly larger than 1,050 K at the interfaces between the core and cladding, and one could assume that the core could be considered as being at the same temperature. This assumption cannot hold for the cladding since its temperature varied about 100 K along its thickness.

**Figure 8.**
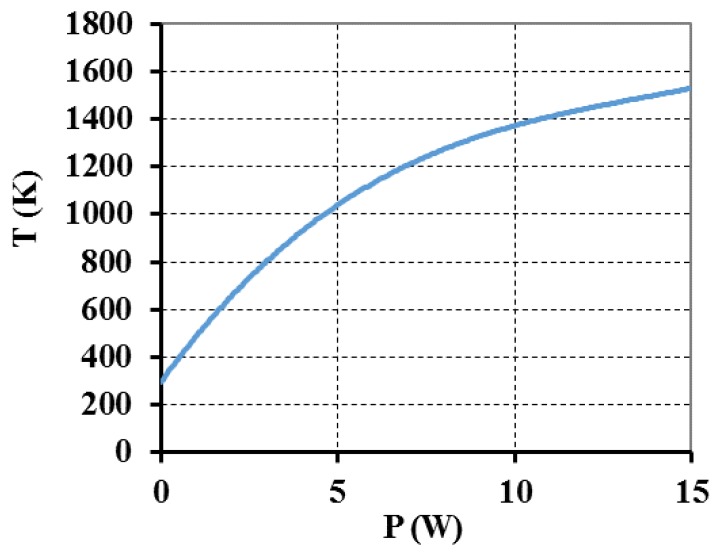
Plot of temperature variation with incident laser power simulated at *x* = *y* = *z* = 0 m. (*τ* = 0.6 s; *F* = 0.461 N).

As the laser power increases, the temperature increases. Figure 8 shows the variation of temperature at the upper surface (at *x* = *y* = 0 m) of the optical fiber with laser power as obtained from the model.

**Figure 9.**
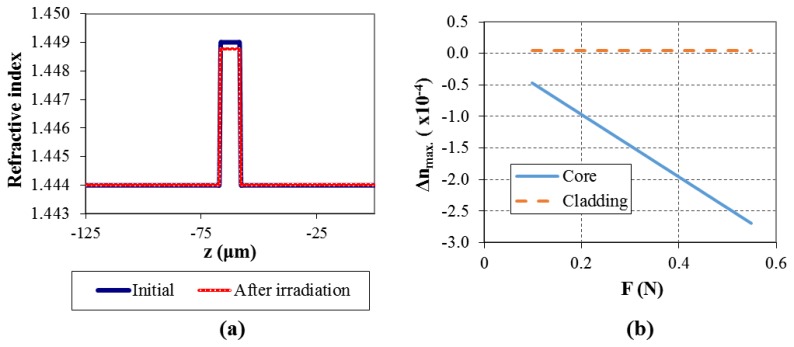
Calculated (**a**) refractive index profiles of the fiber, before and after laser irradiation (*F* = 0.461 N); and (**b**) refractive index change (maximum change for core and cladding) for different applied draw tensions. (*P* = 6 W; *τ* = 0.6 s).

**Figure 10.**
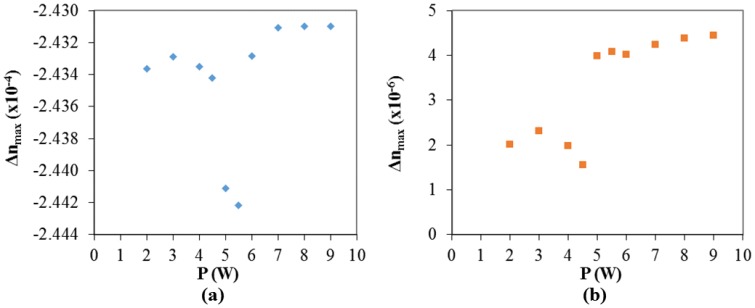
Calculated (maximum) refractive index change at the (**a**) core and (**b**) cladding for different applied laser powers. (τ = 0.6 s; F = 0.461 N).

For the considered example, at *t* = 0.6 s, axial residual thermal stresses values along z-axis were determined as having a maximum of about −0.8 MPa. Axial elastic stresses act in the opposite direction and were calculated as being *σ_x_*_,_*_co_* = 35 MPa and *σ_x_*_,_*_cl_* = 0.153 MPa. The resulting refractive index change (the difference between final and initial values) is calculated to be in the order of −2 × 10^−4^ for the core and 4 × 10^–6^ for the cladding. The refractive index distribution in the fiber (along the *z*-axis), before and after the laser irradiation is showed in Figure 9a and the maximum refractive index change (core and cladding) for different draw forces is plotted in Figure 9b. Figure 10 shows the calculated (maximum) refractive index change at the core and cladding for different laser power. Under the considered conditions, even if the refractive index of the core shows minor changes by increasing the laser power, it is possible to observe the beginning of the contribution of thermal stresses around 5 W. This is clearly observed regarding the refractive index change for the cladding, where a well-defined step occurs between 4.5 W and 5 W.

**Figure 11.**
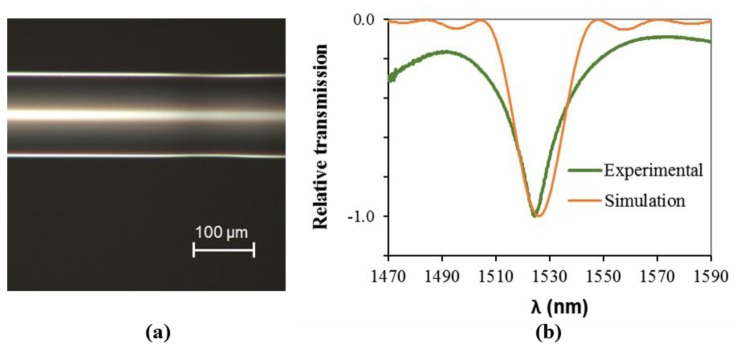
(**a**) Picture showing an irradiated zone from a 25 mm LPFG with 500 μm period and (**b**) experimentally obtained and simulated relative normalized spectral transmission. (*P* = 6 W; *τ* = 0.6 s; *F* = 0.461 N).

Figure 11a shows a microscope photograph of an irradiated fiber under the conditions considered in this work. The imaged zone was part of a 25-mm grating with a period of 500 μm, and the visible affected area along the fiber's axis was about 130 μm (supported on several measurements along the grating). Also visible was a (small) micrometric deformation of the fiber. Figure 11b shows the spectral transmission of the resultant LPFG, comparing the experimental data with the simulated spectrum. The latter was obtained using the refractive index changes obtained by the FEM model (and mentioned before) and using a simulation tool developed by Baptista [34] based on the three layer model developed by Erdogran [35,36].

Analyzing both theoretical and experimental data, besides the relative spectral transmission data agreement, one can consider that it is necessary to reach temperatures higher than 1,000 K to accomplish an LPFG under these conditions. This assumption relies on the cross-analysis between the visible affected length of the fiber (around 130 μm) and the fiber's back surface temperature for *x* = −65 μm or *x* = 65 μm. This is in accordance with experimental evidences that, even for higher power conditions for which tapering occurs, the laser focal dimension influence on the affected volume prevail, at least at the surface [37].

Also, although we simulated the impact of different laser powers and weights, experimentally, using lower laser powers (typically <5 W) no LPFGs were obtained. For higher laser powers (typically >8 W) or higher weights (typically >60 g, *F* > 0.588 N) tapering occurs, a phenomena not included in the developed model. These experimental observations can also be inferred from results obtained in the FEM model, based on the analysis of the plots shown in Figure 10, in particular regarding Figure 10b. In the latter case, and based on the analysis previously made, it is easily identified the required minimum applied power (5W) for writing LPFG under the considered conditions.

The values obtained by the model are also in agreement with those estimated by other authors for the refractive index modulations necessary for achieving a fiber optic grating. Temperatures obtained by the model are similar to those obtained by other authors for arc-induced LPFG (e.g., in the range 1,100 K–1,400 K according to [27]) and the refractive index changes obtained are also within the overall range mentioned (in the order of magnitude of 10^−4^ for the core and 10^−6^ for the cladding) in other works [17,19–21,27]. The obtained behavior of the refractive index change as the applied drawing force increases complies with the most recent experimental indications that the refractive index of the core decreases while the opposite occurs in the cladding, and that this change occurs primarily in the core [20,21].

Nevertheless, due to the complexity of the physical phenomena involved, refractive index change dependence on stress also requires further research. Future work should focus on experimental measurements of temperature, stresses and refractive index changes induced by laser radiation. Although published works can contribute in assessing the validity of the results, the influence of specific characteristics of the fibers is a well-recognized issue. In particular, the effect of pre-existing stresses (e.g., from the fiber manufacture), differences in the materials, or other unaccounted phenomena can influence the performance of the FEM model when compared with real data. Similarly, the influence of the experimental data uncertainties on the model must be analyzed in detail, as well as the impact of the several approximations considered (e.g., transverse stresses are neglected), unaccounted phenomena like eventual changes on the glass polarizability and using standard material data.

## 5. Conclusions

The FEM model presented in this work demonstrated its potential to simulate the thermo-mechanical processes involved in writing LPFGs using CO_2_ laser radiation. It takes in account the influence of temperature on the most relevant thermal and mechanical parameters of the fiber material, as well as convective and radiative effects. The model is 3D, considers a focused laser line irradiating a single-mode fiber for a given duration, and generates temperature and residual stresses distributions and the required refractive index change. An example of irradiating a single-mode optical fiber was presented and both theoretical (simulated) and experimental data are analyzed. Although additional work should be performed to further validate the analysis done (mainly regarding stresses acting in the optical fiber), the FEM results are in accordance with literature and experimental data.

## Figures and Tables

**Table 1.** Geometry data and mesh statistics.

	**Central Geometry**		**External Geometries**	
	
	**Cladding**	**Core**	**Cladding**	**Core**
Geometry				
Length (mm)	0.7	0.7	5.15 × 2	5.15 × 2
Radius (μm)	62.5	4.1	62.5	4.1

Mesh (tetrahedral)				
# elements	24,122	1,231	27,917	1,704
min. quality	0.0474	0.1357	0.2347	0.2017
volume ratio	9.46 × 10^−4^	0.0934	0.0019	0.4914
